# Delamination Localization in CFRP Laminates Using One-Way Mixing of Ultrasonic Guided Waves

**DOI:** 10.3390/s26061912

**Published:** 2026-03-18

**Authors:** Maoxun Sun, Yuheng Liu, Longfei Li, Xinyu Zhang, Biao Xiao, Yue Zhang, Hongye Liu

**Affiliations:** 1 School of Mechanical Engineering, University of Shanghai for Science and Technology, Shanghai 200093, China; sunmaoxun@usst.edu.cn (M.S.); woaislg2290165484@163.com (Y.L.); 13863846383@163.com (L.L.); 2 School of Mechanical Engineering, Inner Mongolia University of Science and Technology, Baotou 014010, China; zxy_lhb@163.com; 3 Shanghai Institute of Special Equipment Inspection and Technical Research Co., Ltd., Shanghai 200062, China; 4 School of Mechanical Engineering, Nantong University, Nantong 226019, China; yuezhang@ntu.edu.cn; 5 School of Optical-Electrical and Computer Engineering, University of Shanghai for Science and Technology, Shanghai 200093, China; liuhongye@usst.edu.cn

**Keywords:** Nonlinear ultrasonics, CFRP laminates, One-way mixing of guided waves, Delamination localization

## Abstract

**Highlights:**

**What are the main findings?**

- The one-way mixing of A_0_ and S_0_ modes generates both difference-frequency components (A_0_ modes) and sum-frequency components (A_0_ and A_1_ modes), which propagate along both the forward and backward directions.
- Delamination in CFRP laminates is successfully localized using one-way mixing of ultrasonic guided waves by adjusting the corresponding time delay.

**What are the implications of the main findings?**

- Ultrasonic signals can be excited and received on the same sides, and be applied in buried plate-like or pipe-like structures.
- We offer an efficient approach for the early damage detection and accurate damage localization of buried plate-like and pipe-like structures.

**Abstract:**

Carbon fiber-reinforced polymer (CFRP) laminates are widely used in aircraft skins due to their advantages of high strength and lightweight properties. However, their laminate structure and energy-absorbing characteristics result in low-energy impact damage, such as delamination, that is often invisible but can lead to catastrophic failure. Consequently, early detection of delamination in CFRP laminates is necessary. Nonlinear ultrasonic guided waves exhibit high sensitivity to delamination, and second harmonics are widely employed. Compared to second harmonics, one-way mixing of ultrasonic guided waves can excite and receive signals simultaneously at the same location, thereby precisely localizing delamination. This capability has the potential for inspecting buried structures. However, existing literature has not yet fully addressed the generation mechanism of one-way mixing in CFRP laminates nor its interaction with delamination. Based on finite element simulation, this study investigates one-way mixing of A_0_ modes and S_0_ modes in CFRP laminates. Utilizing pulse-inversion techniques and two-dimensional fast Fourier transforms, the modes and propagation directions of difference-frequency components and sum-frequency components are determined. Furthermore, by utilizing the normalized acoustic nonlinearity parameter χ’ and adjusting the position of the mixing zone through different time delays, delamination in the CFRP laminate is successfully localized.

## 1. Introduction

Carbon fiber-reinforced polymer (CFRP) is widely utilized in aeronautical, aerospace, automotive, and marine sectors due to its superior properties, including a high strength-to-weight ratio, high stiffness, and excellent corrosion resistance [1,2,3]. Due to the laminated and energy-absorbing characteristics of skins fabricated from anisotropic CFRP, low-energy impact damage (including delamination, fiber or matrix cracking) caused by factors such as tool dropping may not be readily apparent to the naked eye [4], yet internal damage may already exceed design damage tolerance limits. Delamination is a common component of barely visible impact damage (BVID) [5]. If engineers fail to promptly locate and address delamination through maintenance or replacement, these defects will progressively propagate, ultimately leading to catastrophic failure of CFRP laminates. Consequently, the early detection of delamination within carbon fiber-reinforced composites is of paramount importance [6]. Non-destructive testing of CFRP laminates primarily employs eddy current [7], thermography [8], optical fiber [9], bulk wave [10,11], and ultrasonic guided wave [6,12,13,14,15] techniques. Compared to other non-destructive testing methods, ultrasonic waves can penetrate CFRP laminates to detect internal defects or damage. Compared to NDT based on bulk ultrasonic waves, ultrasonic guided wave-based NDT offers advantages such as a broader detection range and higher efficiency [16,17]. Traditional guided ultrasonic waves can only detect defects or damage whose dimensions are comparable to the wavelength. Traditional ultrasonic guided wave NDT techniques struggle to effectively detect defects or damage when their dimensions are less than half the wavelength, or when the acoustic impedance differs little from that of the surrounding medium [18]. However, nonlinear ultrasonic guided waves exhibit heightened sensitivity to microstructural changes such as dislocations, precipitates, micro-cracks, and delaminations [19]. They can generate higher harmonics, static components, and mixing components [20]. Nonlinear ultrasonic guided waves in plate-like structures caused by material nonlinearities are associated with damages like fatigue [21], creep [22], plastic deformation [22], and thermal aging [23], etc. Other typical damages, like micro-cracks and delaminations, also induce nonlinear guided wave responses, but their generation mechanisms are fundamentally different from the aforementioned cases. It has been proven that contact acoustic nonlinearity (CAN) can reveal the nonlinear interaction of nonlinear guided waves (including the second harmonics, nonlinear mixing of guided waves, etc.) in plate-like structures and damages like micro-cracks and delamination by many researchers [13,19,24].

Nonlinear ultrasonic guided waves interact with microstructural discontinuities to generate higher-order harmonics at frequencies distinct from the fundamental wave. Among these, the second harmonic of ultrasonic guided waves has found relatively widespread application [25]. Rauter et al. [26] investigated CFRP laminates with a stacking sequence [0]_4_ and demonstrated that the fundamental S_1_ mode generates a cumulative second harmonic (S_2_ mode). The relative acoustic nonlinearity parameter is more sensitive to impact damage than linear acoustic parameters, such as group velocity. Soleimanpour and Ng [14], utilizing three-dimensional finite element modeling and experimental measurements, concluded that contact acoustic nonlinearity in composites primarily originates from delamination. Furthermore, compared to higher harmonics of antisymmetric modes, those of symmetric modes exhibit greater sensitivity to delamination. Tie et al. [15] employed integrated finite element analysis and experimental measurements to detect barely visible impact damage induced by low-velocity impacts in a [0/90]_5_ CFRP laminates. Their findings indicate that both the second harmonic amplitude and the relative acoustic nonlinear parameters increase as impact energy and delamination area increase. However, nonlinearities within the measurement system (including instruments, sensors, and couplant) may also generate second harmonics, complicating the identification of their true source [19,25,26,27]. Furthermore, the measured second harmonic represents an average across the entire tested area, precluding the precise localization of delamination within CFRF laminates.

Ultrasonic guided wave mixing offers flexibility in selecting the mode, frequency, and propagation direction for fundamental, difference-frequency, and sum-frequency components, thereby partially overcoming the limitations of second harmonic generation [17,28]. Based on the propagation directions of fundamental waves a and b, ultrasonic guided wave mixing can be categorized into collinear and non-collinear modes [29,30]. For isotropic materials, both collinear and non-collinear ultrasonic guided wave mixing have been employed for early-stage damage assessment. Cho et al. [31] utilized two-way mixing of the SH_0_ mode to generate the sum-frequency components of the S_0_ mode, enabling the detection of localized fatigue damage in aluminum plates. Aslam et al. [32] used numerical simulations and experimental measurements to evaluate micro-cracks in a 1.6 mm thick aluminum plate via two-way mixing of S_0_ and A_0_ modes. Metya et al. [23] employed the one-way mixing of S_0_ modes to assess local deformation during the creep process of 9Cr-1Mo steel. Deng and Li et al. [33] employed mixing of the L_0_ mode and L_0_ mode to generate cumulative effects of L_0_ modes, evaluating thermally induced local damage in the bending region of L-shaped bends. Blanloeuil et al. [24] utilized non-collinear mixing of SH_0_ modes to detect contact nonlinearity related micro-defects or micro-damage, such as cracks and delamination. Guan et al. [34] revealed the forward scattering intensity increases significantly as crack length grows, while it decreases as crack width and burial depth increase. Li et al. [35] found that the acoustic nonlinear parameter increases linearly with quadratic nonlinearity but monotonically with the size of the mixing zone. Ding al. [36] found that under specific conditions, S_0_ and A_0_ mode waves can interact within a micro-cracked region to generate a resonant A_0_ mode wave. The aforementioned literature primarily focuses on guided wave mixing in isotropic materials. CFRP laminates constitute multilayer structures composed of anisotropic materials. Lan et al. [5] employed piezoelectric transducers and a laser Doppler vibrometer to excite chirp signals and receive sum-frequency components, utilizing chirp guided wave mixing to evaluate delamination and barely visible impact damage with an energy of 16 J. Xie et al. [37] employed an experimental approach to investigate the one-way mixing of fundamental S_0_ and SH_0_ waves in composite laminates, demonstrating its specific sensitivity to impact damage and inherent quadratic nonlinearity over delamination. It should be noted that one-way mixing of ultrasonic guided waves enables simultaneous excitation and reception of acoustic signals at the same location, offering the potential to inspect buried plate-like or pipe-like structures [38]. However, the existing literature has not clarified the mechanism of one-way mixing of ultrasonic guided waves in CFRP laminates, nor the interaction mechanism between mixing and delamination damage. To address the above issues, it is assumed that ultrasonic guided waves propagate in the CFRP laminates without attenuation and dispersion, and that the only nonlinear source, i.e., the contact nonlinearity, is considered at the delamination interface. We investigate one-way mixing of ultrasonic guided waves in CFRP laminates, successfully localizing delamination damage. This research achieved numerical simulation of one-way mixing of ultrasonic guided waves within CFRP laminates. Subsequently, employing pulse-inversion techniques and two-dimensional fast Fourier transforms, the modes and propagation directions of both the difference-frequency and sum-frequency components are determined. Finally, based on the one-way mixing of ultrasonic guided waves, the location of delamination damage was successfully identified.

The remainder of this paper is organized as follows. Section 2 reviews the fundamental theories of ultrasonic guided waves in CFRP laminates, the mixing of ultrasonic guided waves caused by contact nonlinearity, and the principles of damage localization. Section 3 introduces the modeling of ultrasonic guided wave mixing and delamination interaction in numerical simulations, along with the arrangement of receiving points. Section 4 presents the numerical results, detailing the generation and propagation characteristics of the difference-frequency and sum-frequency components. Section 5 provides a comprehensive discussion of these findings, interpreting the specific wave modes and directivity, and demonstrates the determination of the delamination location using the acoustic nonlinearity parameter. Section 6 concludes the research.

## 2. Theoretical Background of the Interaction Between Ultrasonic Guided Wave Mixing and Delamination in CFRP Laminates

### 2.1. Ultrasonic Guided Waves in CFRP Laminates

In this study, a Cartesian coordinate system is established for the CFRP laminates, with the origin positioned on the left side of the laminates. The x-axis and y-axis are defined on the upper surface of the CFRP laminates, with the x-axis parallel to the propagation direction of the ultrasonic guided waves and the y-axis perpendicular to it. The z-axis is oriented along the thickness direction of the CFRP laminates. For a single-layer CFRP laminate where ultrasonic guided waves propagate along the fiber direction, the corresponding equation of motion is expressed as

(1) $$\sigma_{ik,k}=\rho {\ddot{u}}_{i}$$

where *σ* denotes stress, *u* represents displacement, and *ρ* is density. For anisotropic materials, the constitutive equation is formulated, and the geometric equation is established as follows

(2) $$\sigma_{\mathrm{ik}}=C_{\mathrm{iklm}}\varepsilon_{\mathrm{lm}}$$

(3) $$\varepsilon_{\mathrm{lm}}=u_{l,m}+u_{m,l}/2$$

where *ε* denotes strain and C*_iklm_* represents elastic constants. By combining Equations (1)–(3), the wave equation for ultrasonic guided waves in CFRP laminates can be expressed as

(4) $$\rho {\ddot{u}}_{i}=C_{\mathrm{iklm}}u_{i,\mathrm{km}}$$

Assume expressions for *u_x_*, *u_y_*, and *u_z_* are given by

(5) $$\left\{\begin{array}{c} u_{x}=U_{x}e^{[ik(x+\alpha z-c_{p}t)]}\\ u_{y}=U_{y}e^{[ik(x+\alpha z-c_{p}t)]}\\ u_{z}=U_{z}e^{[ik(x+\alpha z-c_{p}t)]} \end{array}\right.$$

where U*_x_*, U*_y_*, and U*_z_* denote the amplitudes of *u_x_*, *u_y_*, and *u_z_*, respectively, *k* is the wavenumber, *c*_p_ is the phase velocity, and α = *k_z_*/*k_x_* (*k_x_* and *k_z_* being the wave numbers of the ultrasonic guided waves displacement along the *x* and *z* axes). By substituting Equation (5) into Equation (4) yields the Christoffel equation.

**Figure 1:**
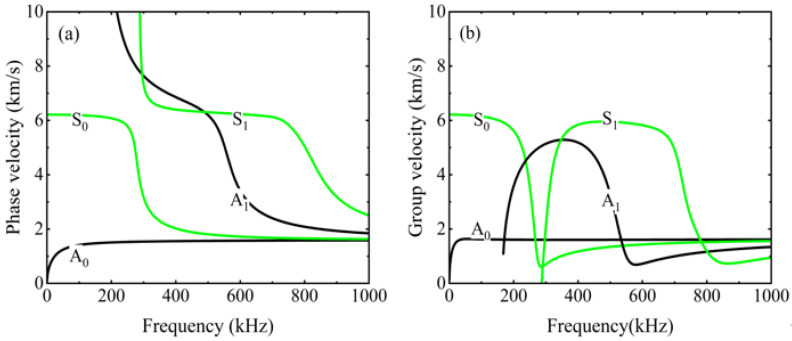
(**a**) Phase velocity dispersion curve and (**b**) group velocity dispersion curve of CFRP laminates.

Since carbon fiber composites can be approximated as transversely isotropic materials, the Christoffel equation can be simplified as

(6) $$\left[\begin{array}{ccc} A_{11} & 0 & A_{13}\\ 0 & A_{22} & 0\\ A_{13} & 0 & A_{33} \end{array}\right]\left[\begin{array}{c} U_{1}\\ U_{2}\\ U_{3} \end{array}\right]=\left[\begin{array}{c} 0\\ 0\\ 0 \end{array}\right]$$

where $A_{11}=C_{11}+C_{55}\alpha^{2}-\rho c_{p}^{2}$, $A_{13}=(C_{13}+C_{55})\alpha$, $A_{22}=C_{66}+C_{44}\alpha^{2}-\rho c_{p}^{2}$ and $A_{33}=C_{55}+C_{33}\alpha^{2}-\rho c_{p}^{2}$. The relationship between elastic constants C*_iklm_* and engineering constants can be expressed as

(7) $$\begin{array}{c} C_{11}=\left[\begin{array}{c} \left(1-v_{23}^{2}\right)/\Delta \end{array}\right]E_{1}\\ C_{12}=C_{13}=\left[v_{12}\left(1+v_{23}\right)/\Delta \right]E_{1}\\ C_{22}=C_{33}=\left[\begin{array}{c} 1-v_{12}^{2}\left(E_{2}/E_{1}\right)/\Delta \end{array}\right]E_{2}\\ C_{23}=\left[\begin{array}{c} v_{23}+v_{12}^{2}\left(E_{2}/E_{1}\right)/\Delta \end{array}\right]E_{2}\\ C_{44}=G_{23}=E_{2}/\left[2\left(1+v_{23}\right)\right]\\ C_{55}=C_{66}=G_{12} \end{array}$$

where $\Delta =1-\nu_{23}^{2}-2\nu_{12}^{2}\left(1+\nu_{23}\right)\left(E_{2}/E_{1}\right)$. The subscripts 1, 2, and 3 denote the material principal directions of the CFRP laminate, direction 1 represents the fiber direction, direction 2 represents the in-plane transverse direction perpendicular to the fibers within the plate, and direction 3 represents the thickness direction of the plate. Since both the top and bottom surfaces of the CFRP laminates are traction-free, we have

(8) $$\sigma_{zx}{|}_{z=0\;\mathrm{or}\;h}=\sigma_{\mathrm{zy}}{|}_{z=0\;\mathrm{or}\;h}=\sigma_{zz}{|}_{z=0\;\mathrm{or}\;h}=0$$

where *h* denotes the thickness of the CFRP laminates. For multilayer structures, the interface continuity condition must be satisfied

(9) $$\left\{\begin{array}{c} u_{x}^{n}{|}_{z=h^{\left(n\right)}}=u_{x}^{(n-1)}{|}_{z=h^{(n-1)}}\\ u_{y}^{n}{|}_{z=h^{\left(n\right)}}=u_{y}^{(n-1)}{|}_{z=h^{(n-1)}}\\ u_{z}^{n}{|}_{z=h^{\left(n\right)}}=u_{z}^{(n-1)}{|}_{z=h^{(n-1)}} \end{array}\right.$$

(10) $$\left\{\begin{array}{c} \sigma_{zx}^{n}{|}_{z=h^{\left(n\right)}}=\sigma_{zx}^{(n-1)}{|}_{z=h^{(n-1)}}\\ \sigma_{zy}^{n}{|}_{z=h^{\left(n\right)}}=\sigma_{zy}^{(n-1)}{|}_{z=h^{(n-1)}}\\ \sigma_{zz}^{n}{|}_{z=h^{\left(n\right)}}=\sigma_{zz}^{(n-1)}{|}_{z=h^{(n-1)}} \end{array}\right.$$

where *h*^(*n*−1)^ denotes the interface between the n th and *n* − 1 th layers in the multilayer structures. By simultaneously solving Equations (6)–(9) in conjunction with the global matrix method, the dispersion curves for the S_0_, A_0_, S_1_, and A_1_ modes in the multilayer CFRP laminates are obtained, as shown in Figure 1.

### 2.2. Contact Acoustical Nonlinearity of Guided Wave Mixing

The delamination damage areas in the CFRP laminates show stiffness asymmetry under ultrasonic guided wave excitation. Ultrasonic guided waves passing through these regions produce nonlinear effects related to contact acoustic nonlinearity, and the relationship between stress and strain can be expressed as a segmented function as follows

(11) $$\sigma =\sigma^{L}+\sigma^{\mathrm{NL}}$$

Among them, the linear part and the nonlinear part are, respectively,

(12) $$\sigma^{L}=C^{\mathrm{II}}\varepsilon (t)$$

(13) $$\sigma^{\mathrm{NL}}=C^{\mathrm{II}}H(\varepsilon -\varepsilon^{0})(\Delta C/C^{\mathrm{II}})\varepsilon (t)$$

where H is the Heaviside unit step function, ε^0^ is the initial static contact strain, and C^II^ is the effective stiffness in the layered closed state. $\Delta C=[C^{\mathrm{II}}-{\left(d\sigma /d\varepsilon \right)}_{\varepsilon >0}]$ represents the difference caused by layered expansion, which decreases stiffness. The existing studies have systematically examined higher-order harmonics arising from acoustic nonlinearities. However, for CFRP laminates and shell structures, there remains a need for further in-depth theoretical analysis of the difference- and sum-frequency components. This article sets $\varepsilon (t)=\varepsilon_{0}\cos\omega_{a}t+\varepsilon_{0}\cos\omega_{b}t$, $\sigma^{\mathrm{NL}}$ equal to

(14) $$\left\{\begin{array}{cc} \sigma^{\mathrm{NL}}=C^{\mathrm{II}}(\Delta C/C^{\mathrm{II}})(\varepsilon_{0}\cos\omega_{a}t+\varepsilon_{0}\cos\omega_{b}t) & 0\text{<}|t|\text{<}\tau_{1}\;\mathrm{and}\;\tau_{2n}\text{<}|t|\text{<}\tau_{2n+1}\\ \sigma^{\mathrm{NL}}=0 & \tau_{2n-1}\text{<}|t|\text{<}\tau_{2n} \end{array},\;n\in N^{+}\right.$$

where $\varepsilon_{0}\cos\omega_{a}\tau_{i}+\varepsilon_{0}\cos\omega_{b}\tau_{i}=\varepsilon^{0}$, $0\text{<}\tau_{i}\text{<}T/2,i\in N^{+}$, among equal to $1/\gcd(f_{1},f_{2})$, $\gcd(f_{1},f_{2})$ represents the greatest common divisor of *f*_1_ and *f*_2_. According to the fast Fourier transform, the spectrum of $\sigma^{\mathrm{NL}}=C^{\mathrm{II}}(\Delta C/C^{\mathrm{II}})(\varepsilon_{0}\cos\omega_{a}t+\varepsilon_{0}\cos\omega_{b}t)$ can be expressed as

(15) $$F(\sigma^{\mathrm{NL}})=\frac{1}{2}[F_{c}(\omega +\omega_{a})+F_{c}(\omega -\omega_{a})+F_{c}(\omega +\omega_{b})+F_{c}(\omega -\omega_{b})]$$

where $F_{c}(\omega )=F_{c}[C^{\mathrm{II}}(\Delta C/C^{\mathrm{II}})]$ equals

(16) $$\begin{array}{l} F_{c}(\omega )=C^{\mathrm{II}}\frac{\Delta C\varepsilon_{0}}{C^{\mathrm{II}}}[2\tau_{1}\sin c(\omega \tau_{2})-2\tau_{2}\sin c(\omega \tau_{2})+2\tau_{3}\sin c(\omega \tau_{3})-2\tau_{4}\sin c(\omega \tau_{4})+\ldots +\\ 2\tau_{2n}\sin c(\omega \tau_{2n})-2\tau_{2n+1}\sin c(\omega \tau_{2n+1})] \end{array}$$

Therefore, the spectrum of the higher-order harmonics can be written as

(17) $$\begin{array}{l} F^{\mathrm{NL}}(l\omega_{a}\pm m\omega_{b})=\frac{1}{2}\left\{F_{c}[(l+1)\omega_{a}\pm m\omega_{b}]\right.+F_{c}[(l-1)\omega_{a}\pm m\omega_{b}]+\\ F_{c}[l\omega_{a}\pm (m+1)\omega_{b}]+F_{c}[l\omega_{a}\pm (m-1)\omega_{b}]\text{\}} \end{array}$$

This study mainly focuses on the difference-frequency components and the sum-frequency components in CFRP laminates, specifically when l = 1 and m = 1.

### 2.3. Adjustment of the Mixing Zone for Delamination Localization

**Figure 2:**
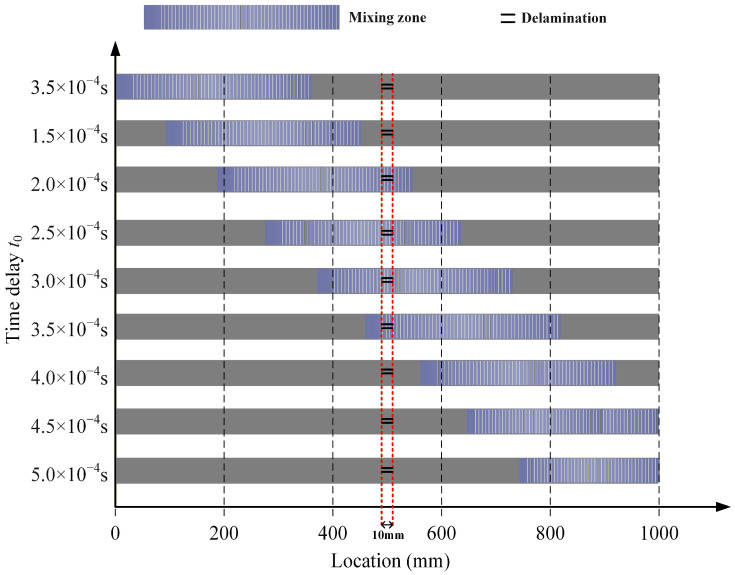
Changing the time delay of the fundamental wave b to adjust the position of the mixing zone.

According to the non-zero power flux principle, the nonlinear mixing of symmetric and antisymmetric modes can produce difference-frequency components or sum-frequency components of the antisymmetric mode. This study separately selects the fundamental wave a of the A_0_ mode and the fundamental wave b of the S_0_ mode. When two fundamental waves interact during delamination, ultrasonic guided wave mixing occurs, generating difference-frequency components and sum-frequency components. This study changes the time delay of the fundamental wave b, sets a fixed step size, gradually shifts the mixing zone backward, and achieves scanning of the CFRP laminates, as shown in Figure 2. The difference-frequency or sum-frequency components are received at the front end of the CFRP laminates, allowing analysis of the extent and location of delamination. Therefore, this study can both excite and receive acoustic signals at the same location, potentially enabling the detection of buried CFRP laminates.

## 3. Numerical Simulations of the Interaction Between Delamination and Ultrasonic Guided Wave Mixing in CFRP Laminates

### 3.1. Model Construction and Parameter Selection

**Figure 3:**
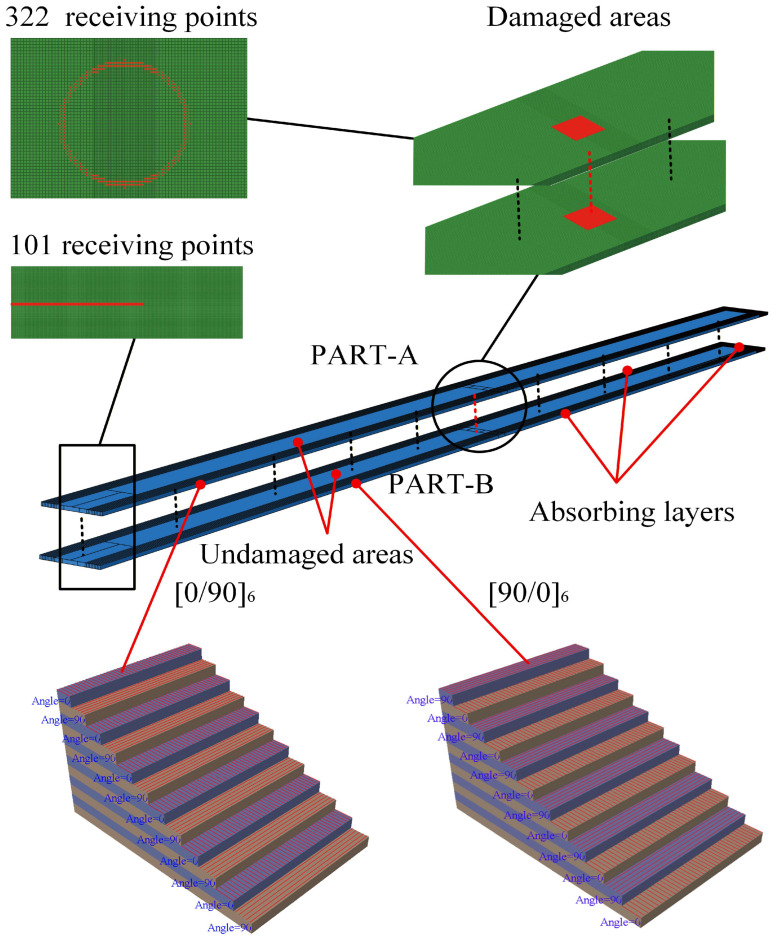
Schematic of the 3D finite element model for CFRP laminates, illustrating the locations of damaged and undamaged areas, absorbing layers, and the arrangements of linear (101 points) and circular (332 points) receiving arrays.

To investigate the occurrence of ultrasonic guided wave mixing in CFRP laminates, the propagation of difference-frequency components or sum-frequency components, and the interaction between delamination and ultrasonic guided wave mixing, we employ numerical simulations using commercial software Abaqus 2021/EXPLICIT. The CFRP laminates are made of the T300/7901, with detailed properties in Table 1, which is assumed as a transversely isotropic material. Two CFRP laminates with dimensions of 1000 mm × 40 mm × 2.4 mm, the stacking sequences of which are [0/90]_6_ and [90/0]_6_. They are recorded as Part A and Part B, respectively. Part A and Part B are combined into a single laminate using Tie constraints to ensure continuity of stress and displacement. Consequently, the symmetrical lay-up of the assembled laminates exhibits is [0/90]_6s_. The CFRP laminates are divided into three regions, i.e., the damaged area, the undamaged area, and the absorbing layers. The absorbing layers comprise three sections with dimensions of 1000 mm × 10 mm × 4.8 mm, 1000 mm × 10 mm × 4.8 mm, and 40 mm × 20 mm × 4.8 mm, respectively. The damaged areas are located in the center of the laminates, with a dimension of 10 mm × 10 mm, as shown in Figure 3. Delamination is introduced between Part A and Part B. The friction formulation for the tangential behavior at the delamination interface is set as frictionless, while the pressure overclosure for normal behavior is set to “hard” contact [34,39]. For the absorbing layer, we no longer neglect the damping of T300/7901. To suppress boundary reflections, Rayleigh damping is introduced in the absorbing regions. In this study, the energy dissipation is implemented through the mass-proportional damping coefficient *a*(*x*), which can be written as [40]

(18) $$\alpha (x)=\alpha_{max}X{\left(x\right)}^{p}$$

where α_max_ denotes the maximum Rayleigh damping coefficient, *X*(*x*) represents the normalized position function, and n is the power-law exponent (typically *p =* 3). The absorbing layers effectively weaken the energy of propagating ultrasonic guided waves, thereby reducing the influence of boundary reflections. The areas between the damaged areas and the absorbing layers are undamaged areas.

According to Equations (19) and (20), the maximum element size (∆*I*) and time step (∆*t*) can be calculated [41]

(19) $$\Delta I=\frac{\lambda_{\min}}{20}$$

(20) $$\Delta t=\frac{1}{20f_{\max}}$$

*λ*_min_ denotes the shortest wavelength of the ultrasonic guided waves, while *f*_max_ represents their maximum frequency. The element size for the undamaged area and absorbing layers is 0.5 mm, whereas the damaged area has a refined element size of 0.25 mm. The CFRP laminates are discretized using a structured meshing technique along the thickness from the bottom surface to the top surface. Element shape and element type are selected as SC8R and Hex, respectively.

**Figure 4:**
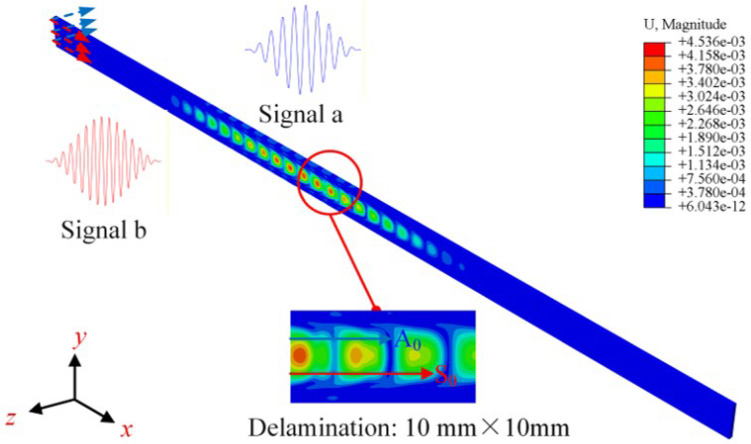
Snapshots of the contour of the displacement field from FE models illustrating the interaction between ultrasonic guided wave mixing and delamination in CFRP laminates.

In this study, displacements with amplitudes of 1 × 10^−5^ m are applied tangentially and normally along the left end face to excite the fundamental modes a and b. The excitation signals for fundamental modes a and b are a 10-cycle Hanning-windowed sinusoidal tone burst at 100 kHz and a 15-cycle Hanning-windowed sinusoidal tone burst at 150 kHz, respectively. The excitation signal for fundamental mode b is time-delayed by *t*_0_. The outer surfaces of the CFRP laminates are treated as free surfaces. At *t*_0_ = 3 × 10^−4^ s and *t* = 4 × 10^−4^ s, the contour plots of the correlated displacement field are shown in Figure 4 below.

### 3.2. Secondary Development of Abaqus/EXPLICIT and Extraction of Time-Domain Signals at Specific Nodes

**Figure 5:**
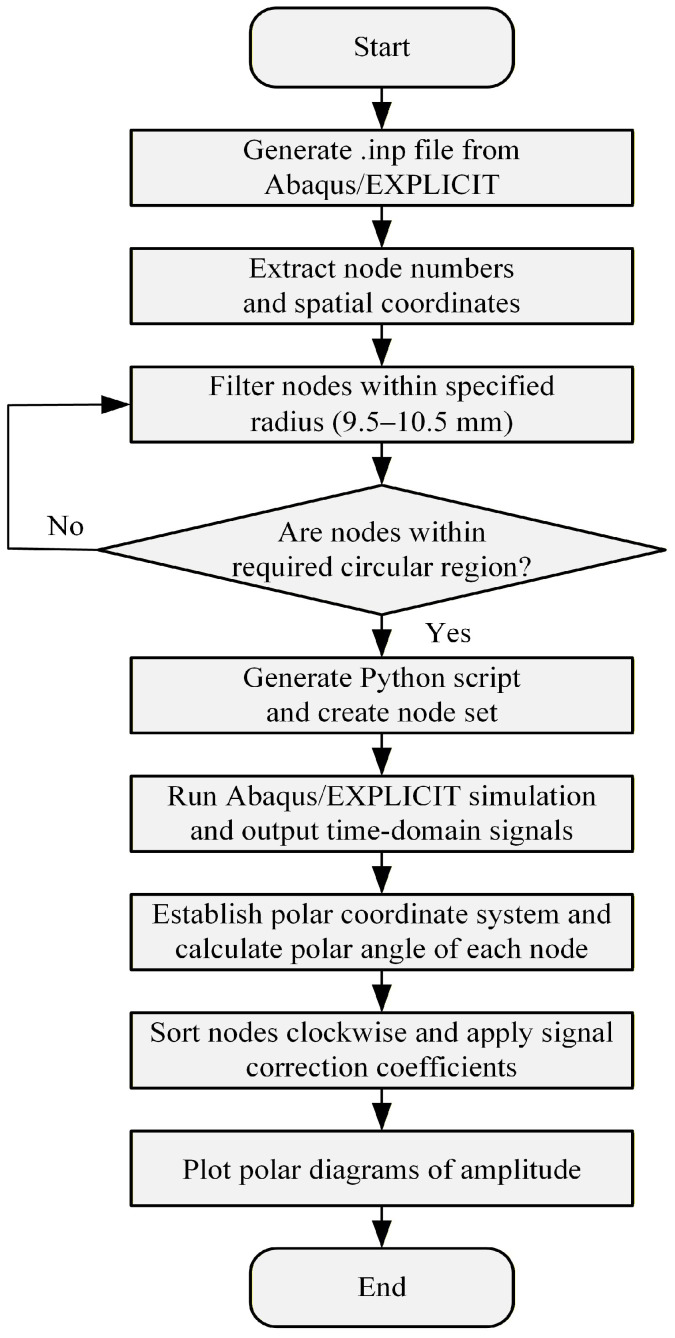
Flowchart for Abaqus/EXPLICIT secondary development.

This study employs Python script files to bypass the graphical interface of Abaqus/EXPLICIT, enabling direct interaction with the Abaqus/EXPLICIT software kernel. The process for submitting script files to Abaqus/EXPLICIT is as follows: first, the Python scripts are interpreted by the built-in Abaqus Python engine to define the finite element model programmatically. Subsequently, the Abaqus/EXPLICIT kernel is triggered to execute the script command, and the model definitions are then generated into an .inp file. Finally, the analysis is submitted to the ABAQUS solver. The flowchart is illustrated in Figure 5.

The positions of the receiving points on the CFRP laminates are shown in Figure 3. This study established 101 receiving points along the x-axis on the left side of the CFRP laminates. Utilizing two-dimensional fast Fourier transforms (2D-FFT), the modes of the difference frequency component or the sum frequency component were analyzed. Additionally, centered on the delamination, 332 receiving points were arranged along a circle with a radius of 10 mm to observe the propagation direction of the difference-frequency components or sum-frequency components.

## 4. Results

### 4.1. Generation of One-Way Mixing of Guided Waves in CFRP Laminates

**Figure 6:**
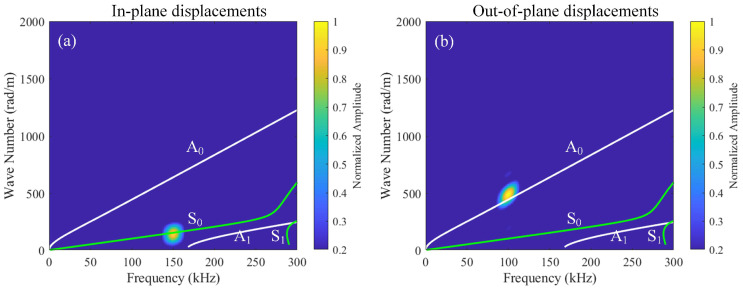
Two-dimensional fast Fourier transforms showing the relationship between normalized amplitude and frequency–wavenumber for (**a**) in-plane displacements (Data Set A) and (**b**) out-of-plane displacements (Data Set B).

**Figure 7:**
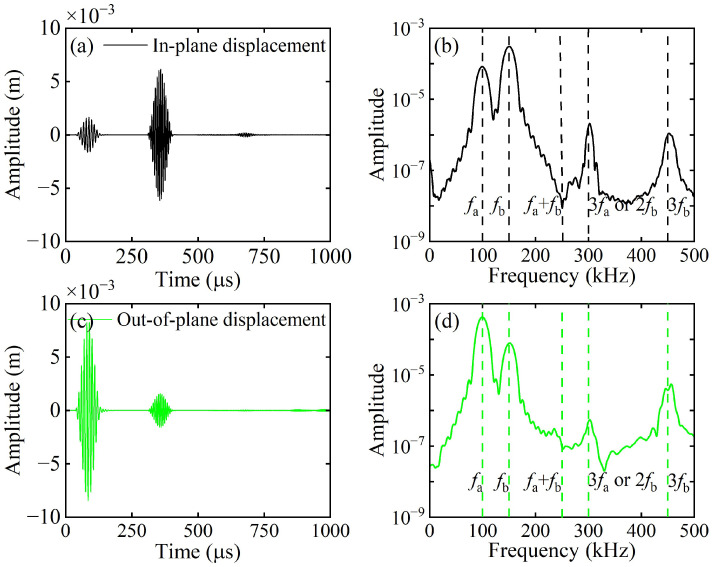
Typical in-plane displacements and their spectra for (**a**,**b**), and out-of-plane displacements and their spectra for (**c**,**d**) when *x* = 50 mm (outside the mixing zone).

A linear array of 101 receiving points is arranged at the left end of the CFRP laminates to capture both in-plane and out-of-plane displacements, denoted as Data Set A and Data Set B, respectively. Both Data Set A and Data Set B are processed by 2D-FFT, and their amplitudes are normalized with respect to the maximum value within the 0–300 kHz frequency band. The normalized results are then combined with the frequency–wavenumber dispersion curves, as illustrated in Figure 6a,b. As shown in Figure 6a, the S_0_ mode at 150 kHz dominates the in-plane displacements on the upper surface. As depicted in Figure 6b, the A_0_ mode at 100 kHz dominates the out-of-plane displacements on the upper surface. Thus, this study excites the fundamental wave a of the A_0_ mode and the fundamental wave b of the S_0_ mode. At *x* = 50 mm (outside the mixing zone), in-plane and out-of-plane displacements were extracted and subjected to fast Fourier transform (FFT). The corresponding time-domain signals and their spectra are shown in Figure 7. In addition to the fundamental waves at *f*_a_ and *f*_b_, it is also observed that the second harmonic of fundamental wave b at 2*f*_b_ (or the third harmonic of fundamental wave a at 3*f*_a_) and the third harmonic of fundamental wave b at 3*f*_b_. However, neither difference-frequency nor sum-frequency components were detected. To validate the aforementioned hypothesis, this study employs pulse-inversion techniques [34,42] to accentuate the difference-frequency and sum-frequency components while eliminating the fundamental waves and second harmonics. The technique is based on the fact that a 180-degree phase reversal changes the algebraic sign of the primary wave, but does nothing to the second harmonic wave. Thus, the ultrasonic measurement can be conducted twice with a 180-degree phase reversal. Thus, summing up the results from these two measurements will cancel the primary waves [42]. In our study, for the first and second models, the excitation signals for fundamental wave a and fundamental wave b have the same phases, set at 0° and 180°, respectively, where Signal 1 and Signal 2 denote the corresponding received signals. In the third model, the excitation signal phase for fundamental wave a is 0°, while that for fundamental wave b is 180°. In the fourth model, the excitation signal phases for fundamental wave a and fundamental wave b are precisely opposite. Signal 3 and Signal 4 denote the corresponding received signals. Finally, the aforementioned time-domain signals are received at the same location and processed according to Equation (21).

(21) $$\left(\mathrm{Signal}\;1+\mathrm{Signal}\;2-\mathrm{Signal}\;3-\mathrm{Signal}\;4\right)/4$$

**Figure 8:**
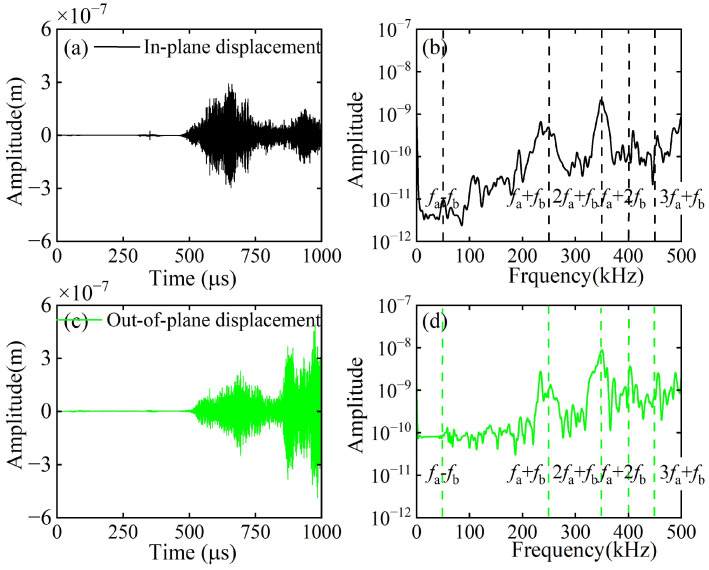
Typical in-plane displacements (**a**,**b**) and their spectra, and out-of-plane displacements (**c**,**d**) and their spectra, processed using the pulse-inversion technique when *x* = 50 mm (outside the mixing zone).

**Figure 9:**
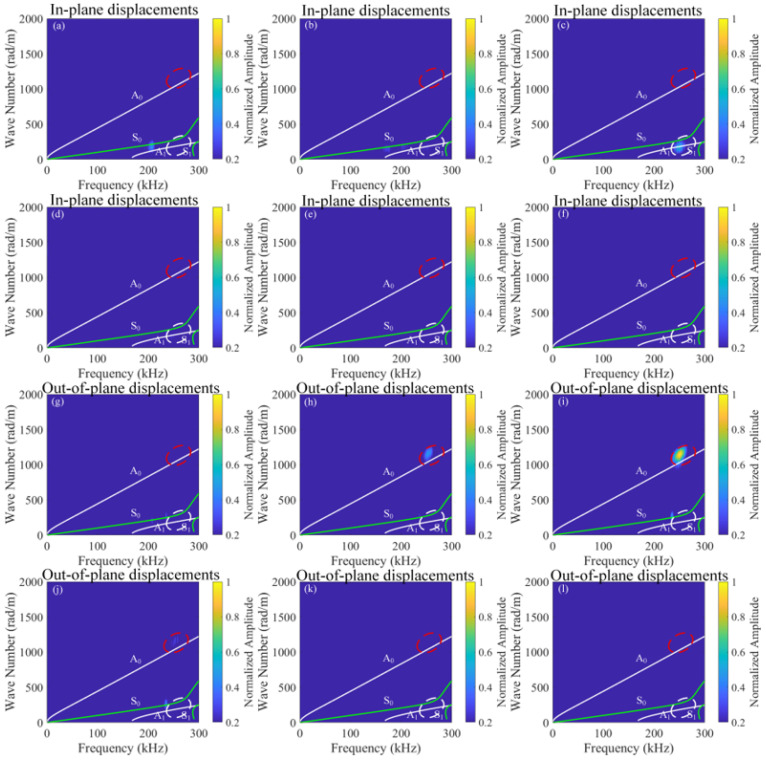
2D-FFT of sum-frequency components for in-plane (**a**–**f**) and out-of-plane (**g**–**l**) displacements at different time delays (*t*_0_), (**a**,**g**) *t*_0_ = 1.5 × 10^−4^ s, (**b**,**h**) *t*_0_ = 2.0 × 10^−4^ s, (**c**,**i**) *t*_0_ = 2.5 × 10^−4^ s, (**d**,**j**) *t*_0_ = 3.0 × 10^−4^ s, (**e**,**k**) *t*_0_ = 3.5 × 10^−4^ s, and (**f**,**l**) *t*_0_ = 4.0 × 10^−4^ s.

Figure 8 presents the representative in-plane and out-of-plane displacements processed via the pulse-inversion techniques at *x* = 50 mm (outside the mixing zone), along with their corresponding spectra. As indicated in Figure 9b,d, the interaction between the ultrasonic guided wave mixing and the delamination generates second-order harmonics at frequencies *f*_b_ − *f*_a_ and *f*_a_ + *f*_b_, third-order harmonics at 2*f*_a_ + *f*_b_ and *f*_a_ + 2*f*_b_, and fourth-order harmonics at 3*f*_a_ + *f*_b_. Hence, this study employs pulse-inversion techniques to identify the modes of both the difference-frequency and sum-frequency components.

**Figure 10:**
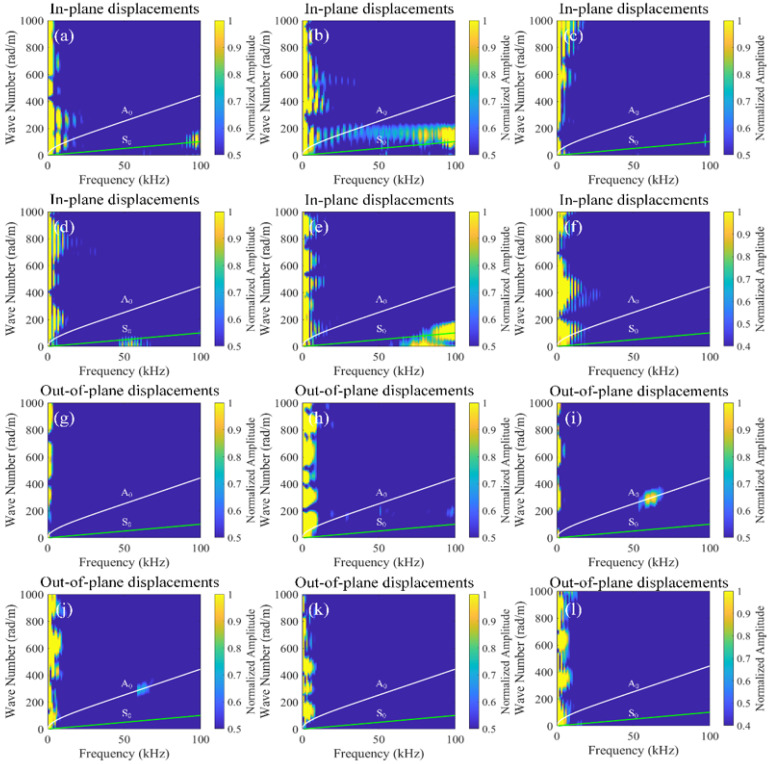
2D-FFT of difference-frequency components for in-plane (**a**–**f**) and out-of-plane (**g**–**l**) displacements at different time delays (*t*_0_), (**a**,**g**) *t*_0_ = 1.5 × 10^−4^ s, (**b**,**h**) *t*_0_ = 2.0 × 10^−4^ s, (**c**,**i**) *t*_0_ = 2.5 × 10^−4^ s, (**d**,**j**) *t*_0_ = 3.0 × 10^−4^ s, (**e**,**k**) *t*_0_ = 3.5 × 10^−4^ s, and (**f**,**l**) *t*_0_ = 4.0 × 10^−4^ s.

To investigate the effect of different time delays, this study establishes 101 receiving points at *x* = 0 mm–50 mm (outside the mixing zone) for different time delays, capturing in-plane and out-of-plane displacements of Signal 1, Signal 2, Signal 3, and Signal 4. These signals are processed using pulse-inversion techniques and 2D-FFT, with the results illustrated in Figure 9 and Figure 10. In the dispersion curves, the green curves represent the symmetric modes, while the white curves denote the antisymmetric modes. In addition, the regions of interest are highlighted in Figure 9. At the frequency of 250 kHz, the A_0_ mode is marked with a red dashed circle, while the A_1_ mode is indicated with a white dashed circle. It is worth noting in Figure 10 that the presence of numerous maxima in the left part is likely caused by the zero-frequency components resulting from the nonlinear interaction between the mixing waves and the delamination damage. Another possibility is that the extreme weakness of the difference-frequency components makes small noise signals appear relatively large.

### 4.2. Propagation Characteristics of Difference-Frequency and Sum-Frequency Components

**Figure 11:**
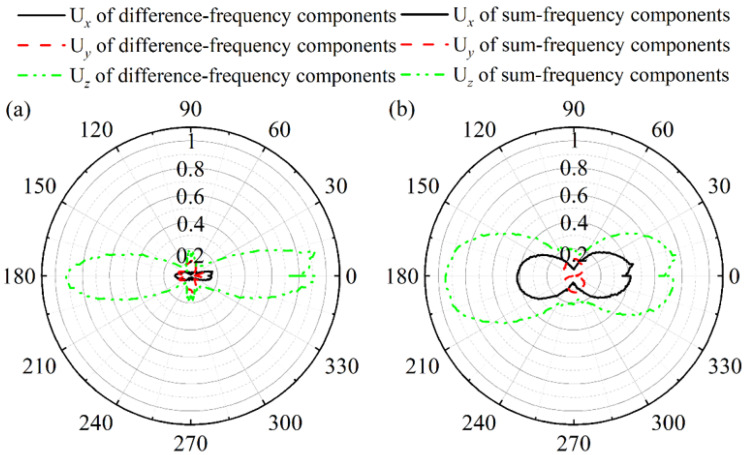
Relationship between the amplitude and polar angle of U*_x_*, U*_y_*, and U*_z_* for (**a**) difference-frequency components and (**b**) sum-frequency components.

To investigate the propagation characteristics of the difference-frequency and the sum-frequency components, a circular array of 332 receiving points is established around the delamination. Displacements along the *x*-axis, *y*-axis, and *z*-axis directions are captured on the upper surface of the CFRP laminates, denoted as U*_x_*, U*_y_*, and U*_z_*, respectively. The relationship between the amplitude and polar angle of U*_x_*, U*_y_*, and U*_z_* for both the difference-frequency components and the sum-frequency components is illustrated in Figure 11. The difference-frequency components correspond to the A_0_ mode, while the sum-frequency components correspond to both the A_0_ and A_1_ modes. As the energy of the A_1_ mode exceeds that of the A_0_ mode, U*_z_* is significantly greater than U*_x_* and U*_y_*, consistent with the wave structures of the A_0_ and A_1_ modes.

**Figure 12:**
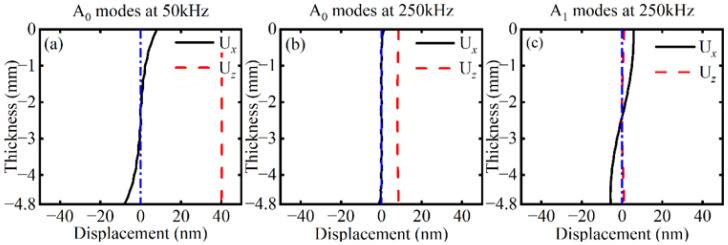
Wave structures of (**a**) the A_0_ mode at 50 kHz, (**b**) the A_0_ mode at 250 kHz, and (**c**) the A_1_ mode at 250 kHz.

As shown in Figure 12, based on the wave structures of the A_0_ and A_1_ modes at 250 kHz, the U*_x_* components of the sum-frequency primarily represent the A_1_ mode, while the U*_z_* components of the sum-frequency primarily represent the A_0_ mode. The maximum amplitude of U*_z_* is significantly larger than that of U*_x_*, implying that the energy of the A_0_ exceeds that of the A_1_ mode. Furthermore, the polar plots of U*_x_* and U*_z_* exhibit a petal-like pattern, with petals directed towards 0° and 180°. Moreover, the U*_y_* patterns for both A_0_ and A_1_ modes show no significant directivity.

### 4.3. Responses of the Acoustic Nonlinearity Parameter

**Figure 13:**
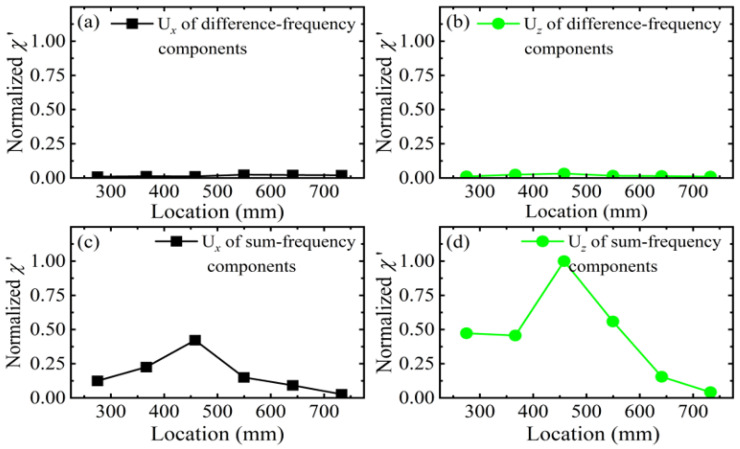
Relationship between the normalized acoustic nonlinearity parameter χ′ and the center position of the mixing zone for difference-frequency components of (**a**) U*^x^* and (**b**) U*^z^*, and sum-frequency components of (**c**) U*^x^* and (**d**) U*^z^*.

This study utilizes the difference-frequency and sum-frequency components from the back-propagating process to determine the x-coordinate of delamination within CFRP laminates. To simplify the analysis, this research does not distinguish between the modes of the difference-frequency and sum-frequency components. The time delay of the fundamental wave b equals *t*_0_, with the mixing zone shifting rearward along the x-axis. The time delays *t*_0_ are set to 1.0 × 10^−4^ s, 1.5 × 10^−4^ s, 2.0 × 10^−4^ s, 2.5 × 10^−4^ s, 3.0 × 10^−4^ s, 3.5 × 10^−4^ s, 4.0 × 10^−4^ s, 4.5 × 10^−4^ s, and 5.0 × 10^−4^ s, respectively. Therefore, this study can adjust the position of the mixing zone to receive in-plane and out-of-plane displacements at *x* = 50 mm (outside the mixing zone). By employing the pulse-inversion technique and fast Fourier transform (FFT), the amplitudes of the difference-frequency and sum-frequency components are extracted to calculate the acoustic nonlinearity parameter χ. This parameter is defined as

(22) $$\chi =\frac{A_{\pm }}{A_{a}A_{b}}$$

where A_±_ represents the amplitude of the difference-frequency and sum-frequency components, and A_a_ and A_b_ denote the amplitudes of fundamental wave a and fundamental wave b, respectively. To enable a scan across the entire CFRP laminates, χ is normalized with respect to its maximum value to yield the normalized acoustic nonlinearity parameter χ’. The relationship between the normalized acoustic nonlinearity parameter χ’ and the center position of the mixing zone is illustrated in Figure 13. Compared to the normalized acoustic nonlinearity parameter χ’ of the difference-frequency component, the normalized acoustic nonlinearity parameter χ’ of the sum-frequency component exhibits greater sensitivity to delamination. Notably, the variation in the normalized acoustic nonlinearity parameter χ’ for U*_z_* is higher than that for U*_x_*.

## 5. Discussion

### 5.1. Mode Identification and Analysis of Difference-Frequency or Sum-Frequency Components

Based on Figure 9 and Figure 10, when 1.5 × 10^−4^ s ≤ *t*_0_ < 2.0 × 10^−4^ s, the interaction region of fundamental wave a and fundamental wave b lies behind the delamination, and no ultrasonic guided wave mixing occurs. When 2.0 × 10^−4^ s ≤ *t*_0_ ≤ 3.5 × 10^−4^ s, the interaction zone of fundamental wave a and fundamental wave b begins to overlap with the delamination, and sum-frequency components emerge as modes A_0_ and A_1_, and difference-frequency components also emerge, specifically the A_0_ mode. Subsequently, the interaction zone of fundamental wave a and fundamental wave b gradually separates from the delamination, and the energy of the difference-frequency components and the sum-frequency components progressively diminishes until it vanishes. When 3.5 × 10^−4^ s < *t*_0_ ≤ 4.0 × 10^−4^ s, the interaction zone of fundamental wave a and fundamental wave b lies ahead of the delamination, and no ultrasonic guided wave mixing occurs. Consequently, the interaction between the guided mixing of A_0_ mode and S_0_ mode with the delamination generates difference-frequency components of A_0_ mode and sum-frequency components of A_0_ and A_1_ modes.

Based on the directivity patterns obtained in Section 4.2, the difference-frequency components of the A_0_ mode, the polar plots of U*_z_* and U*_x_* exhibit a petal-like pattern, indicating that the wave propagates along the carbon fiber with the angle of 0° towards 0° and 180°. Furthermore, the U*_y_* pattern for the A_0_ mode exhibits no significant directivity. This indicates that the difference-frequency components of the A_0_ mode propagate along the *x*-axis, simultaneously in both forward and backward directions.

In summary, the sum-frequency components of both the A_0_ and A_1_ modes propagate simultaneously along the *x*-axis, both forward and backward. Consequently, the interaction between the mixing of the A_0_ mode with the S_0_ mode and the delamination generates both difference-frequency components and sum-frequency components propagating simultaneously along the *x*-axis, both forward and backward.

### 5.2. Localization of Delamination in CFRP Laminates

The observations in Figure 13 reveal the underlying relationship between the mixing zone and the delamination location. Compared to the normalized acoustic nonlinearity parameter χ′ of the difference-frequency component, the normalized acoustic nonlinearity parameter χ′ of the sum-frequency component exhibits greater sensitivity to delamination. When the mixing zone coincides with the delamination, both the difference-frequency component and the sum-frequency component emerge. Furthermore, the closer the center of the mixing zone is to the center of the delamination, the greater the normalized acoustic nonlinearity parameter χ′. Therefore, this study demonstrates the feasibility of determining the x-coordinate of the delamination within the CFRP laminates based on the maximum value of the normalized acoustic nonlinearity parameter χ′ and the position of the mixing zone.

To further validate the rationality of the proposed one-way mixing mechanism and localization strategy, the findings of this study are compared with the existing literature. Xie et al. [37] recently provided valuable experimental insights into one-way mixing in composite laminates. Their results indicated that the S_0_-SH_0_ mode combination is relatively insensitive to delamination under specific resonance conditions, whereas the one-way S_0_-A_0_ mixing method could be more suitable for the detection of delamination damage in composite laminates than the one-way S_0_-SH_0_ mixing method. In contrast, the present work focuses primarily on theoretical modeling and numerical simulation based on a contact acoustic nonlinearity. By establishing finite element model that simulates the behavior at the deamination interface, this study explains the generation process of the components with difference-frequency and sum-frequency. Furthermore, it analyzes the spectra and modes of both difference-frequency and sum-frequency components, elucidating their relationship with the delamination location. In terms of localization strategies, Yu et al. [43] utilized linear Lamb wave features to localize delamination in curved CFRP plates, while Yan et al. [44] developed a UNet++-based data-driven framework for full-wavefield imaging and achieved high localization accuracy. Different from these approaches, the present work explores a physics-based pathway by dynamically tracking the normalized acoustic nonlinearity parameter (χ′) through controlled mixing zones. This strategy directly exploits the contact nonlinearity at the delamination and provides clear physical interpretability. Demiral et al. [45] investigated the formation mechanisms of delamination during machining processes, and Fikry et al. [46] investigated delamination growth. The nonlinear evaluation method proposed in this study complements these efforts by providing an additional diagnostic tool for composite structural integrity assessment. It also offers important practical value for the early detection and prevention of small-scale damage in composite materials.

## 6. Conclusions

This study establishes a physical model based on contact nonlinearity theory to investigate the interaction between one-way mixing of ultrasonic guided waves and delamination in CFRP laminates. Based on finite element simulation, a three-dimensional finite element model is constructed to analyze the generation mechanism, modal characteristics, and propagation behavior of difference-frequency and sum-frequency components, as well as their relationship with delamination location. By incorporating group velocity calculations of the fundamental waves, an analytical relationship between the mixing zone and the delamination position is established, providing a theoretical and numerical foundation for delamination localization. Numerical results demonstrate that contact acoustic nonlinearity at the delamination interface generates difference-frequency components, sum-frequency components, and higher harmonic components. The difference-frequency and sum-frequency components propagate simultaneously along the x-axis in both forward and backward directions with specific modal characteristics. By adjusting the relative time delay between the fundamental waves, the position of the mixing zone can be controlled to scan the structure, and a significant enhancement of mixing components occurs when the mixing zone overlaps with the delamination region. The normalized acoustic nonlinearity parameter χ′ decreases as the distance between the mixing zone center and the delamination center increases, indicating its effectiveness as a quantitative indicator for delamination localization. Since one-way mixing requires that transmitters and receivers be positioned at the same end of the structure, it offers a simplified sensor configuration and shows potential for early detection of micro-defects in buried plate-like or pipe-like composite structures.

## Figures and Tables

**Table 1:** Mechanical properties of T300/7901.

*ρ* (kg/m^3^)	E1 (GPa)	E2 (GPa)	E3 (GPa)	G12 (GPa)	G13 (GPa)	G23 (GPa)	v12	v13	v23
1792	125.9	11.3	11.3	5.43	5.43	3.98	0.3	0.3	0.42

## Data Availability

Data will be made available on request.
